# Supplementary material for: A series of five population‐specific Indian brain templates and atlases spanning ages 6–60 years
Source: Hum Brain Mapp. 2020 Aug 26;41(18):5164–75. doi: 10.1002/hbm.25182 (PMC7670651; doi:10.1002/hbm.25182)
Supplement: Supplementary file 1 — Figure S1 The CNR between GM and WM improves consistently across the successive template creation stages in all the template age‐groups C1‐C5. Figure S2: The five IBTs (C1‐5) with three sets of sagittal, coronal and axial view displayed as underlay in grayscale and edge‐filtered version of the MNI 2009 non‐linear template mask as overlay for size comparison. High tissue contrast and detail are evident in each case. Figure S3: The five population‐average IBTs (C1‐5) with three sets of sagittal, coronal and axial view displayed as underlay in grayscale and the respective typical subject for each IBT version as the overlay. Arrow points to example regions in C1 age‐band regions where the typical version provides greater details than the underlying population‐average version. Figure S4: The five IBTs (C1‐5) with three sets of sagittal, coronal and axial view displayed as underlay in grayscale and the respective Indian maximum probability map version of the DK atlas (FreeSurfer's 2000 Atlas) as overlay in AFNI's “ROI i256” color scale. Figure S5: The five IBTs (C1‐5) with three sets of sagittal, coronal and axial view displayed as underlay in grayscale and the respective Indian maximum probability map version of the Destrieux atlas (FreeSurfer's 2009 Atlas) as overlay in AFNI's “ROI i256” color scale. Figure S6: 3D surface view of the brain atlases for the C1‐IBT age band. The top row shows the maximum probability map (MPM) version of the DK atlas (FreeSurfer's 2000 Atlas) and the bottom row shows MPM version of the Destrieux atlas (FreeSurfer's 2009 Atlas) for the C1 age band. Figure S7: Scatterplot with marginal densigram for pairwise correlations between absolute values of logarithm of the relative volume ratios and mean absolute deformation value across all the regions in the maximum probability map (MPM) version of the DK atlas (FreeSurfer's 2000 Atlas) at each age‐group C1‐C5. Figure S8: Axial views for three example region of interest from MPM‐2000 IBT atlas for a [file HBM-41-5164-s001.pdf]

This section provides supplementary figures and codes to the material in the main text.

The contrast-to-noise ratio (CNR) between GM and WM for successive template creation steps (Figure S1) was calculated as

$$CNR = \frac{|mean(SI_{GM}) - mean(SI_{WM})|}{sqrt(SD_{GM}^2) + sqrt(SD_{WM}^2)} \quad (1)$$

where SI_{GM} / SI_{wM} and SD_{GM}/SD_{WM} are mean signal intensity within the gray and white matter respectively, and the corresponding standard deviations.

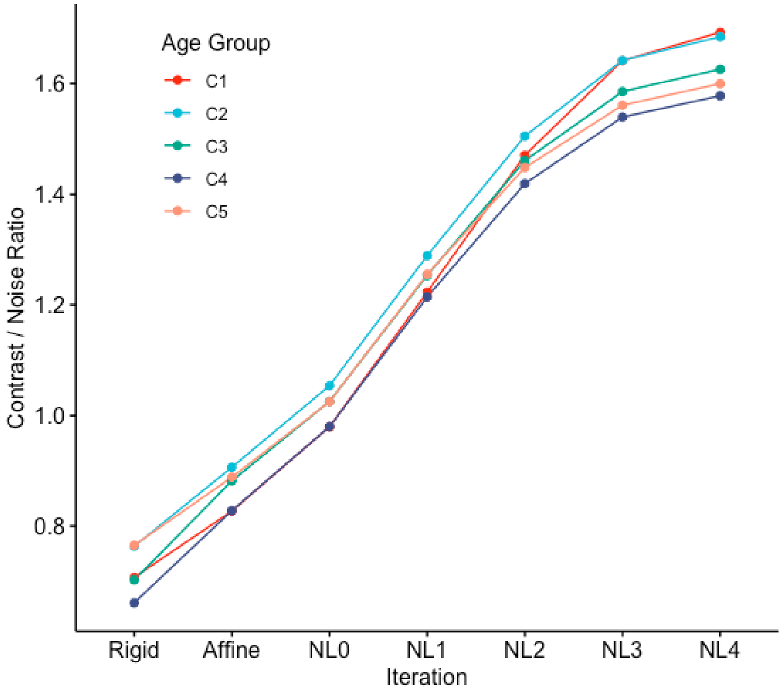

Figure S1: The CNR between GM and WM improves consistently across the successive template creation stages in all the template age-groups C1-C5.

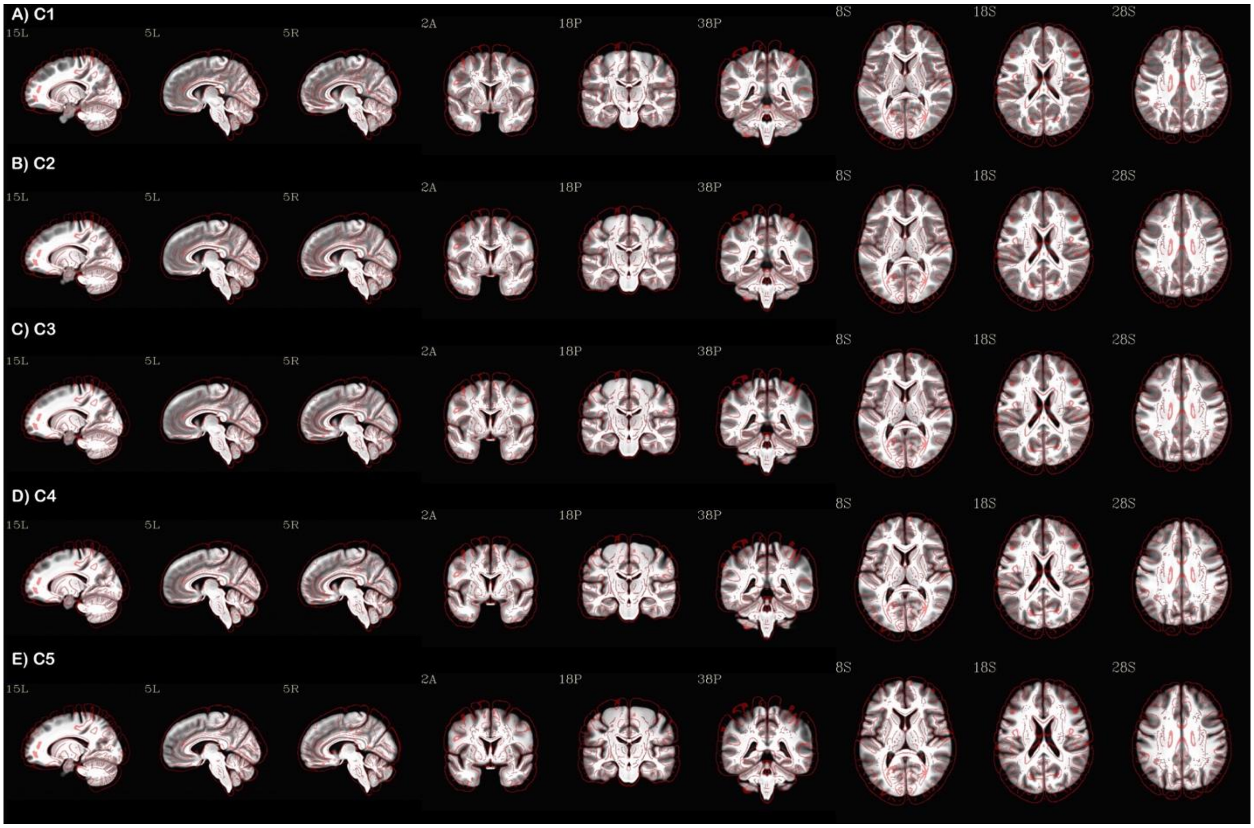

Figure S2: The five IBTs (C1-5) with three sets of sagittal, coronal and axial view displayed as underlay in grayscale and edge-filtered version of the MNI 2009 non-linear template mask as overlay for size comparison. High tissue contrast and detail are evident in each case.

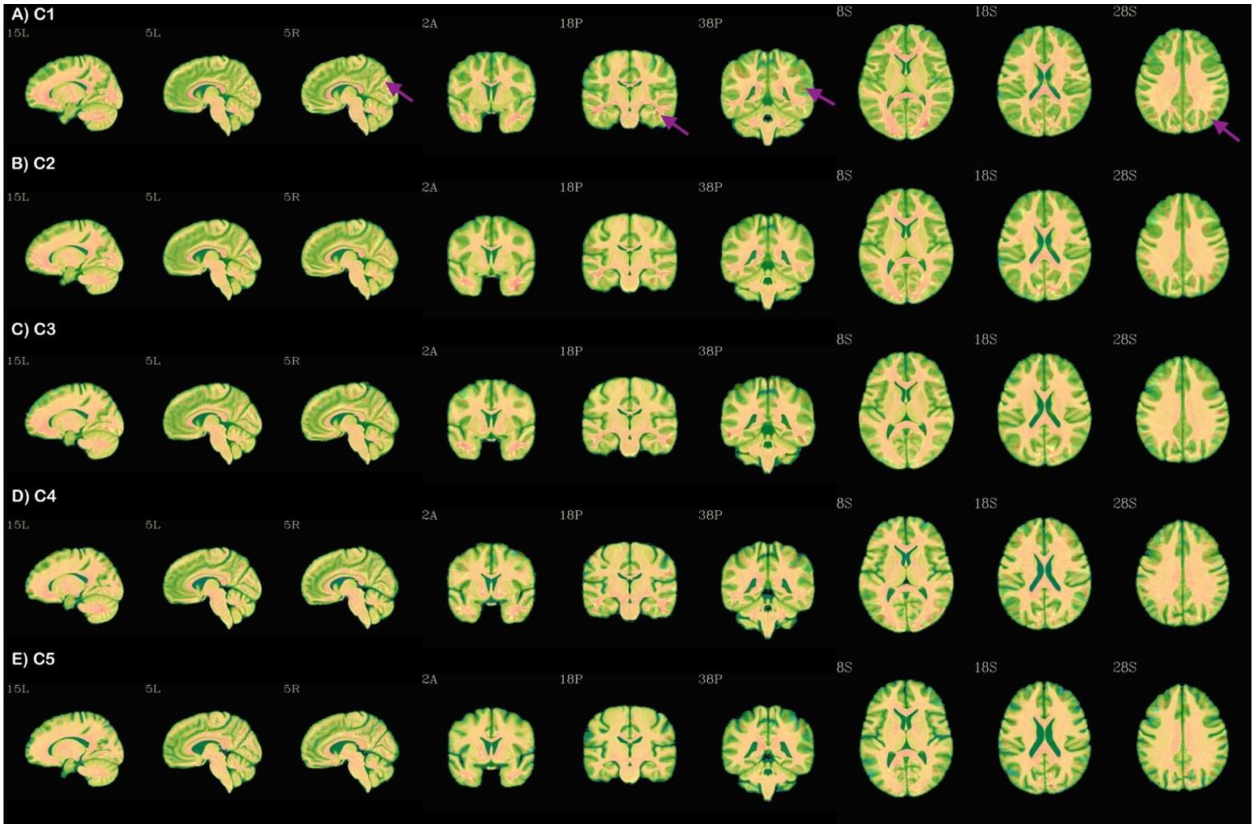

Figure S3: The five population-average IBTs (C1-5) with three sets of sagittal, coronal and axial view displayed as underlay in grayscale and the respective typical subject for each IBT version as the overlay. Arrow points to example regions in C1 age-band regions where the typical version provides greater details than the underlying population-average version.

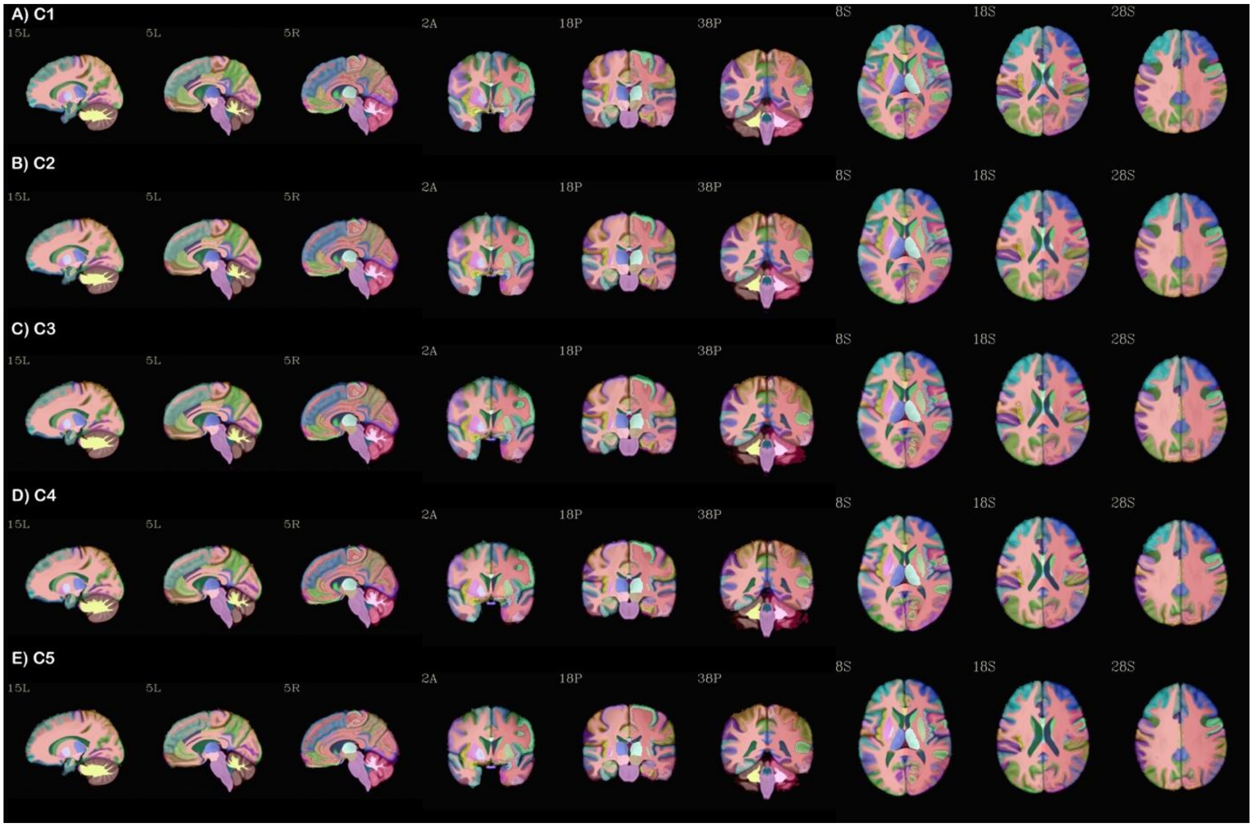

Figure S4: The five IBTs (C1-5) with three sets of sagittal, coronal and axial view displayed as underlay in grayscale and the respective Indian maximum probability map version of the DK atlas (FreeSurfer's 2000 Atlas) as overlay in AFNI's "ROI_i256" color scale.

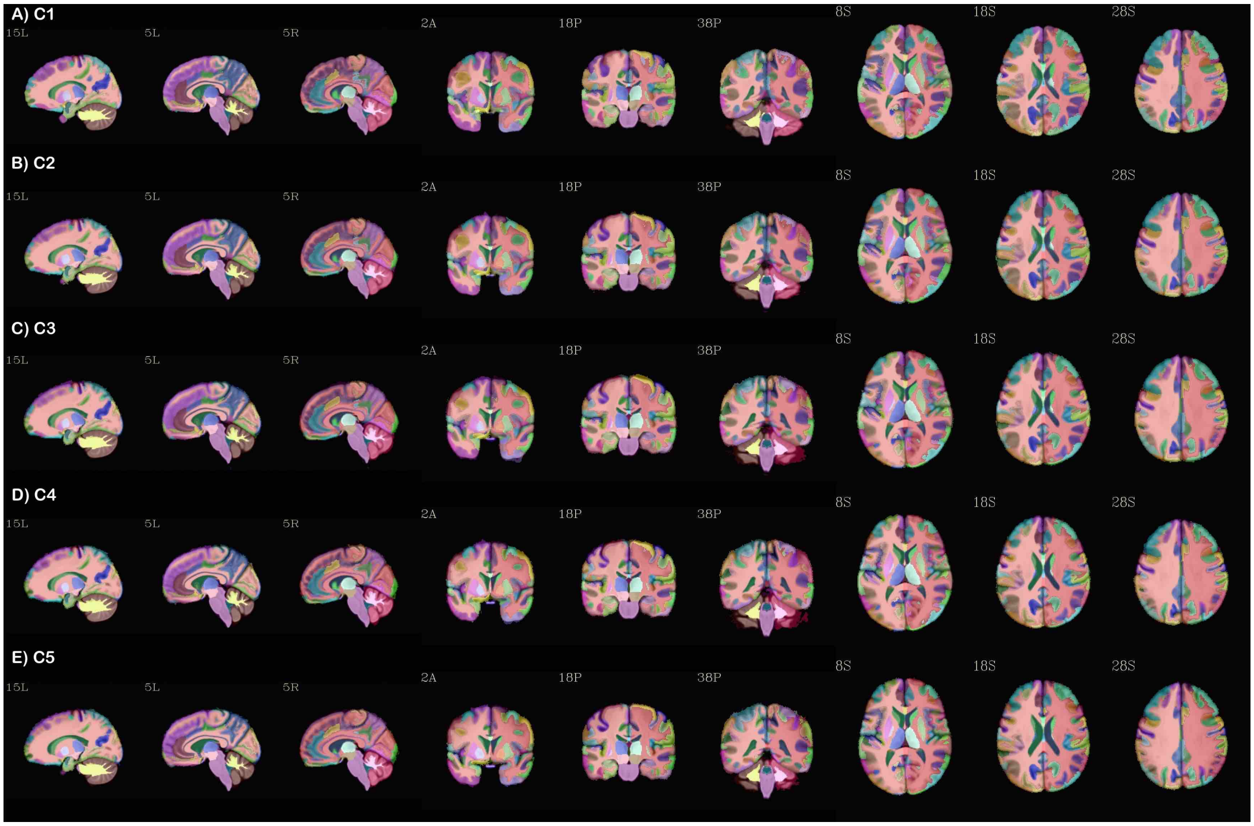

Figure S5: The five IBTs (C1-5) with three sets of sagittal, coronal and axial view displayed as underlay in grayscale and the respective Indian maximum probability map version of the Destrieux atlas (FreeSurfer's 2009 Atlas) as overlay in AFNI's "ROI_i256" color scale.

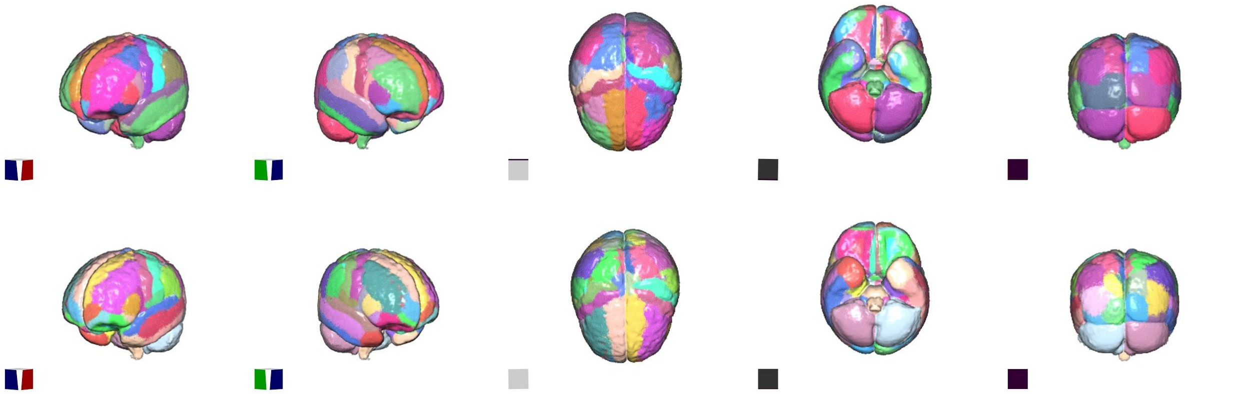

Figure S6: 3D surface view of the brain atlases for the C1-IBT age band. The top row shows the maximum probability map (MPM) version of the DK atlas (FreeSurfer's 2000 Atlas) and the bottom row shows MPM version of the Destrieux atlas (FreeSurfer's 2009 Atlas) for the C1 age band.

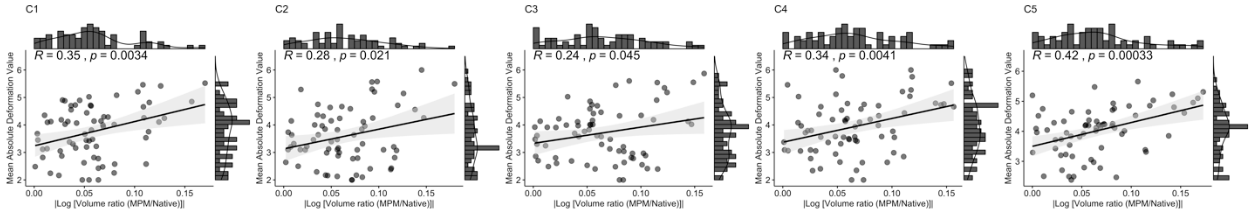

Figure S7: Scatterplot with marginal densigram for pairwise correlations between absolute values of logarithm of the relative volume ratios and mean absolute deformation value across all the regions in the maximum probability map (MPM) version of the DK atlas (FreeSurfer's 2000 Atlas) at each age-group C1-C5.

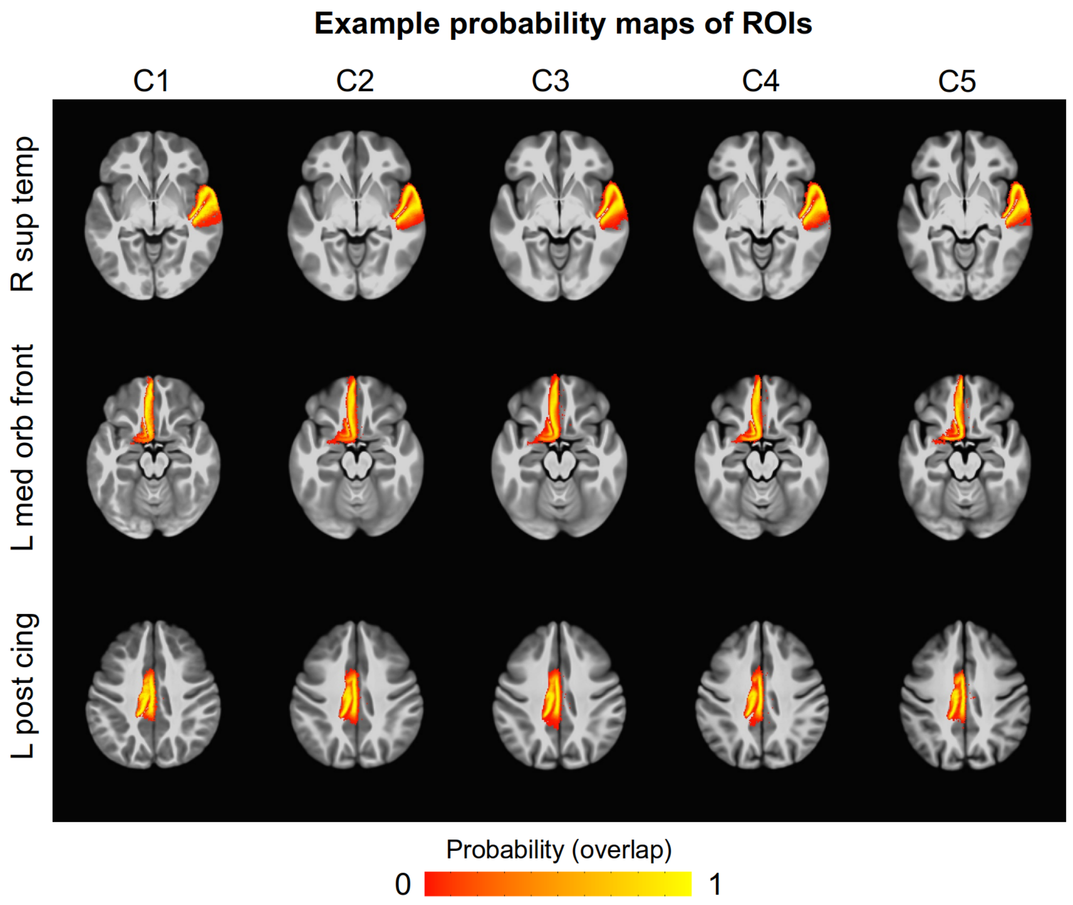

Figure S8: Axial views for three example region of interest from MPM-2000 IBT atlas for all the age groups. The top row shows probability map for right superior temporal gyrus, middle row shows left medial orbital frontal gyrus and the bottom row shows left posterior cingulate gyrus. The color intensity reflects probability density estimates (ranging from 0 to 1)

Supplementary Information:

Example afni_proc.py command for comparing validation tests.

```
#!/bin/bash

subj=$1         # subject ID
topdir=$2       # group level directory for input
outdir=$3       # group level directory for output
btemplate=$4    # brain template name

tpath=`@FindAfniDsetPath ${btemplate}`
sdir=${topdir}/${subj}

\mkdir -p ${outdir}

afni_proc.py                                                      \
    -subj_id          ${subj}                                     \
    -out_dir          ${outdir}/${subj}.results                   \
    -blocks           despike tshift align tlrc volreg mask regress   \
    -copy_anat        ${sdir}/anatSS.${subj}.nii                  \
    -anat_has_skull   no                                          \
    -dsets            ${sdir}/${subj}_rest.nii.gz                 \
    -tcat_remove_first_trs  3                                     \
    -align_opts_aea   -ginormous_move  -deoblique on              \
                      -check_flip  -cost lpc+ZZ                   \
    -mask_epi_anat    yes                                         \
    -volreg_align_to  MIN_OUTLIER                                 \
    -volreg_align_e2a                                             \
    -volreg_tlrc_warp                                             \
    -tlrc_base        ${tpath}/${btemplate}                       \
    -tlrc_NL_warp                                                 \
    -tlrc_NL_warped_dsets                                         \
        ${sdir}/anatQQ.${subj}.nii                                \
        ${sdir}/anatQQ.${subj}.aff12.1D                           \
        ${sdir}/anatQQ.${subj}_WARP.nii                           \
    -volreg_warp_dxyz        3                                    \
    -mask_segment_anat       yes                                  \
    -regress_censor_outliers  0.1                                 \
    -regress_censor_motion    0.3                                 \
    -regress_apply_mot_types  demean deriv                        \
    -regress_est_blur_errts                                       \
    -html_review_style       pythonic                             \
    -execute
```
